# Demonstrative operation of four-terminal memristive devices fabricated on reduced TiO_2_ single crystals

**DOI:** 10.1038/s41598-018-38347-z

**Published:** 2019-02-22

**Authors:** Shotaro Takeuchi, Takuma Shimizu, Tsuyoshi Isaka, Tetsuya Tohei, Nobuyuki Ikarashi, Akira Sakai

**Affiliations:** 1 Graduate School of Engineering Science, Osaka University, 1-3 Machikaneyama-cho, Toyonaka, Osaka 560-8531 Japan; 2 Institute of Materials and System for Sustainability, Nagoya University, Furo-cho, Chikusa, Nagoya 464-8603 Japan; 3 Now at SanDisk Limited, 800 Yamanoisshiki-cho, Yokkaichi, Mie 512-8550 Japan

## Abstract

Resistive switching (RS) was demonstrated in four-terminal planar memristive devices fabricated on reduced TiO_2_ (TiO_2−x_) single crystal substrates. In the device, a pair of diagonally opposing electrode terminals is used to modify the distribution of oxygen vacancies in the region between another pair of diagonally opposing electrode terminals. This allowed microscopic visual observations of the oxygen vacancy distribution based on electrocoloring. The visual contrast observed in the TiO_2−x_ reflects the oxygen vacancy concentration in the electrically active zone of the device, which can be modified by application of various external voltages to the electrodes. The current that flows in the device is significantly dependent on the modified oxygen vacancy distribution and the resultant resistance is switchable when the polarization of the applied external voltage is reversed. The crystallographic orientation of the TiO_2−x_ substrate has a strong influence on the reversible RS phenomenon. Mechanisms behind the voltage-driven resistance change are elaborated with the aid of microscopic analysis for both crystalline and electronic structures in the electrically active zone of the device. Suppression of the formation of irreversible conductive structures comprised of accumulated oxygen vacancies is a key to establishing reversible RS in the device.

## Introduction

Resistive switching (RS) in memristors has attracted a great deal of attention for application to nonvolatile memory devices and neuromorphic computing^1–8^. In one class of memristors, it is typically composed of a metal/transition-metal-oxide/metal structure, in which the transition metal oxide can function as a conductive or resistive layer, depending on the applied voltage or the current injected into the device. The RS properties of memristors are required to be well controlled and the devices must exhibit reliable reversibility. To date, the mechanism for the RS phenomenon has been explained based on the generation and redistribution of oxygen vacancies in the oxide caused by application of an external voltage^2,9–21^. As-fabricated memristors generally have high resistance and often require an electroforming process where a conductive filament is preformed to lower the resistance locally in the oxide. Oxygen vacancies play a key role in modulating the conductive filaments, and their distribution is considered to determine the high or low resistance states (HRS or LRS) of the device. Non-filamentary, homogeneous RS has also been demonstrated based on the bulk ion conductivity of an oxygen-deficient amorphous main-group oxide thin film^22^. The RS behavior has been well explained by considering coupled ion drift and diffusion motion, and the oxygen concentration profile.

Reduced titanium dioxide (TiO_2−x_) is a typical RS material for use in memristors^1,2,10^. The filamentary mechanism is arguably the most commonly accepted explanation for the RS phenomenon in TiO_2−x_^2,23,24^, which is closely related to the local phase transformation that creates oxygen-deficient compounds, such as rutile TiO_2_ crystals with polytype shear plane defects or Magnéli phases in TiO_2_^2,25^. However, with regard to memristors driven by the filamentary RS mechanism, it is frequently argued that the formation of such conductive filaments is rather stochastic, has less controllability, and is more or less accompanied by other physical changes in the device structure, such as blow-off or massive redistribution of electrodes^24–28^, thereby posing a serious problem in the practical use of such devices. Therefore, non-filamentary-type RS operation based on modulation of the bulk carrier conduction is essential for the development of memristors with high stability, reliability, and controllability.

In this study, we demonstrate precise control of the oxygen vacancy distribution and related bulk carrier conduction properties in non-filamentary-type memristive devices with a four-terminal structure fabricated on TiO_2−x_ single crystal substrates. In this device, a pair of diagonally opposing electrodes is used to modify the oxygen vacancy distribution in the electrically active zone between another pair of diagonally opposing electrodes. Although the size of this device is on the order of several tens of micrometers, which is not as small as conventional two-terminal memristors, our strategy is to embrace this size and explore modification of the oxygen vacancy distribution in the electrically active zone of the device. Microscopic visual observations of the oxygen vacancy distribution were conducted based on the electrocoloring effect in TiO_2−x_^29^, in which regions with higher concentrations of Ti^3+^ appear dark blue, indicating the presence of oxygen vacancies^30^. This allows the motion of oxygen vacancies under a DC electric field to be traced during device operation. The correlation between the modulation of the oxygen vacancy distribution in the electrically active zone and the RS properties (HRS and LRS) was investigated.

## Results and Discussion

**Figure 1:**
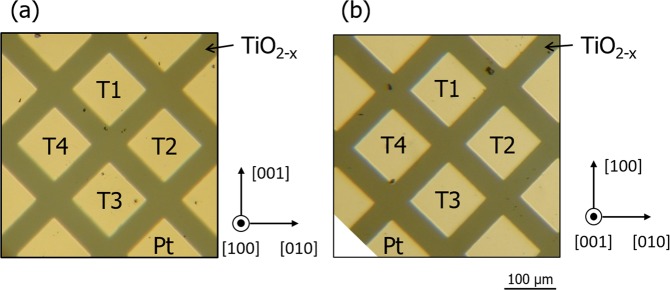
Optical micrographs showing four-terminal planar device structures fabricated on reduced rutile TiO_2−x_ (**a**) (100) and (**b**) (001) surfaces.

Figure 1 shows reflection-mode optical micrographs of our four-terminal memristive devices fabricated on (100) and (001) substrates. The terminals are labeled T1 to T4, with the T2 and T4 terminals being used to modify the oxygen vacancy distribution in the region between the T1 and T3 terminals.

**Figure 2:**
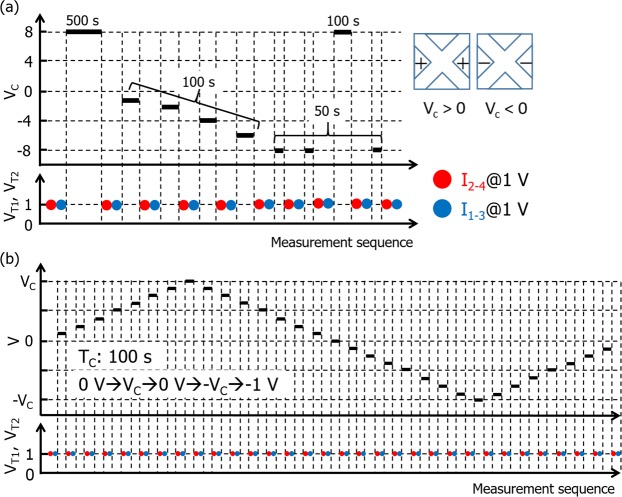
Typical sequences for measurement of RS properties of devices. (**a**) Diagram illustrating the controllability of the oxygen vacancy distribution in the electrically active zone of the devices and (**b**) the reversibility of the RS properties in the devices.

The basic protocol for electrical measurements using the device consisted of write and non-disturbing read voltage application steps. First, the current flow between T1 (T2) and T3 (T4), *I*_1–3_ (*I*_2–4_), was measured at an applied voltage of 1 V to probe the initial state. A constant write voltage *V*_*C*_, was then applied to both T2 and T4 for a certain duration *T*_*C*_, under the condition that both T1 and T3 were grounded. Subsequently, *I*_1–3_ (*I*_2–4_) was again measured at an applied voltage of 1 V to probe the state of the device. This protocol was repeated while the values of *V*_*C*_ and *T*_*C*_ were varied. Figure 2 shows typical sequences of voltage application and current measurements for the device. As a proof-of-concept demonstration to evaluate *V*_*C*_-driven control of the oxygen vacancy distribution, the sequence shown in Fig. 2(a) was employed. On the other hand, the sequence shown in Fig. 2(b) was used to evaluate the repeatability of RS, where the *V*_*C*_ value was varied to generate a ramp-up and ramp-down waveform.

**Figure 3:**
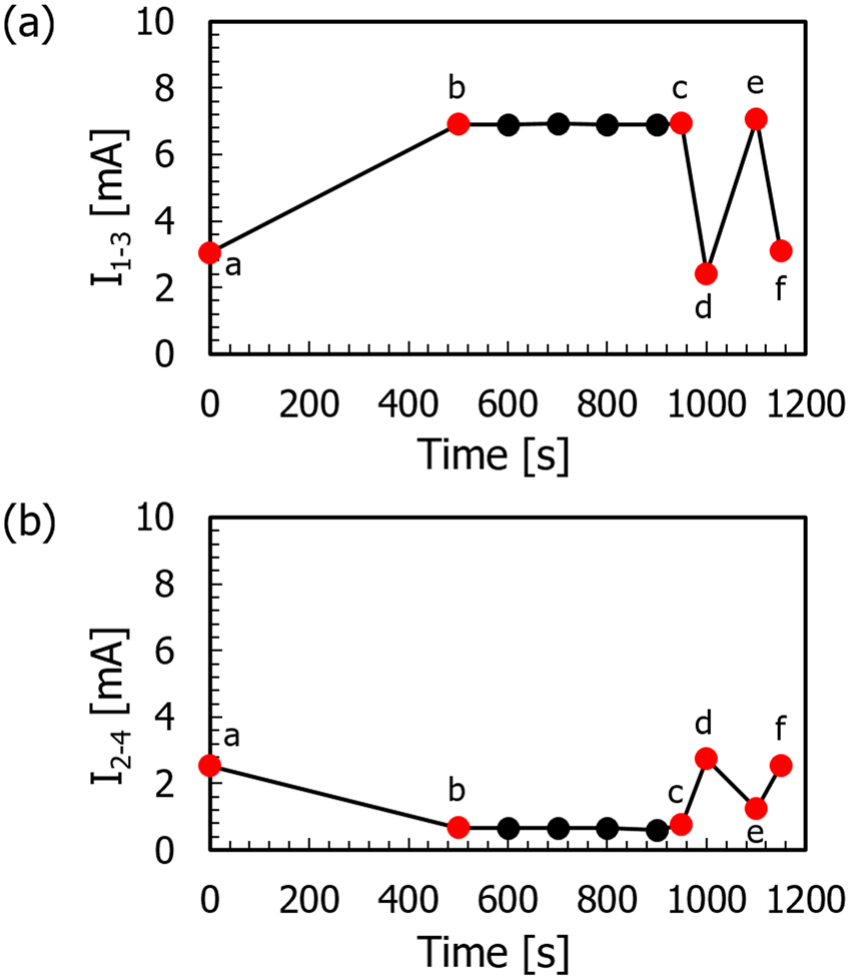
Variations of (**a**) *I*_1–3_ and (**b**) *I*_2–4_ at 1 V as function of cumulative time *T*_*C*_ for application of voltage *V*_*C*_. (Inset) Labels a–f show measurement stage of *I*_1–3_ and *I*_2–4_ at cumulative times of 0, 500, 950, 1000, 1100, and 1150 s, respectively.

We first examined the interplay between the resistance change and the modulation of the oxygen vacancy distribution in the (100) device. Figure 3(a,b) respectively show typical results for the variations of *I*_1–3_ and *I*_2–4_ as a function of the cumulative time *T*_*C*_ for application of the write voltage *V*_*C*_ according to the sequence of Fig. 2(a). The initial values of *I*_1–3_ and *I*_2–4_ were almost the same, as expected; however, after application of *V*_*C*_ = 8 V for 500 s, *I*_1–3_ increased while *I*_2–4_ decreased. This indicates that the resistance in the region between T1 (T2) and T3 (T4) was changed due to the application of *V*_*C*_. Both the current values then remained constant until 950 s, even though the polarity of *V*_*C*_ was changed and the value was negatively and stepwise increased from −1 V to −8 V, as shown in Fig. 2(a). A significant decrease (increase) of *I*_1–3_ (*I*_2–4_) occurred after application of an additional −8 V for 50 s (at cumulative time of 1000 s), and the resultant *I*_1–3_ (*I*_2–4_) value was slightly lower (higher) than the initial value. Subsequent high and low (low and high) values of *I*_1–3_ (*I*_2–4_) were obtained after the application of *V*_*C*_ at +8 and −8 V, respectively, which indicated a reversible change of the resistance in the region between diagonally opposing electrodes in the device.

**Figure 4:**
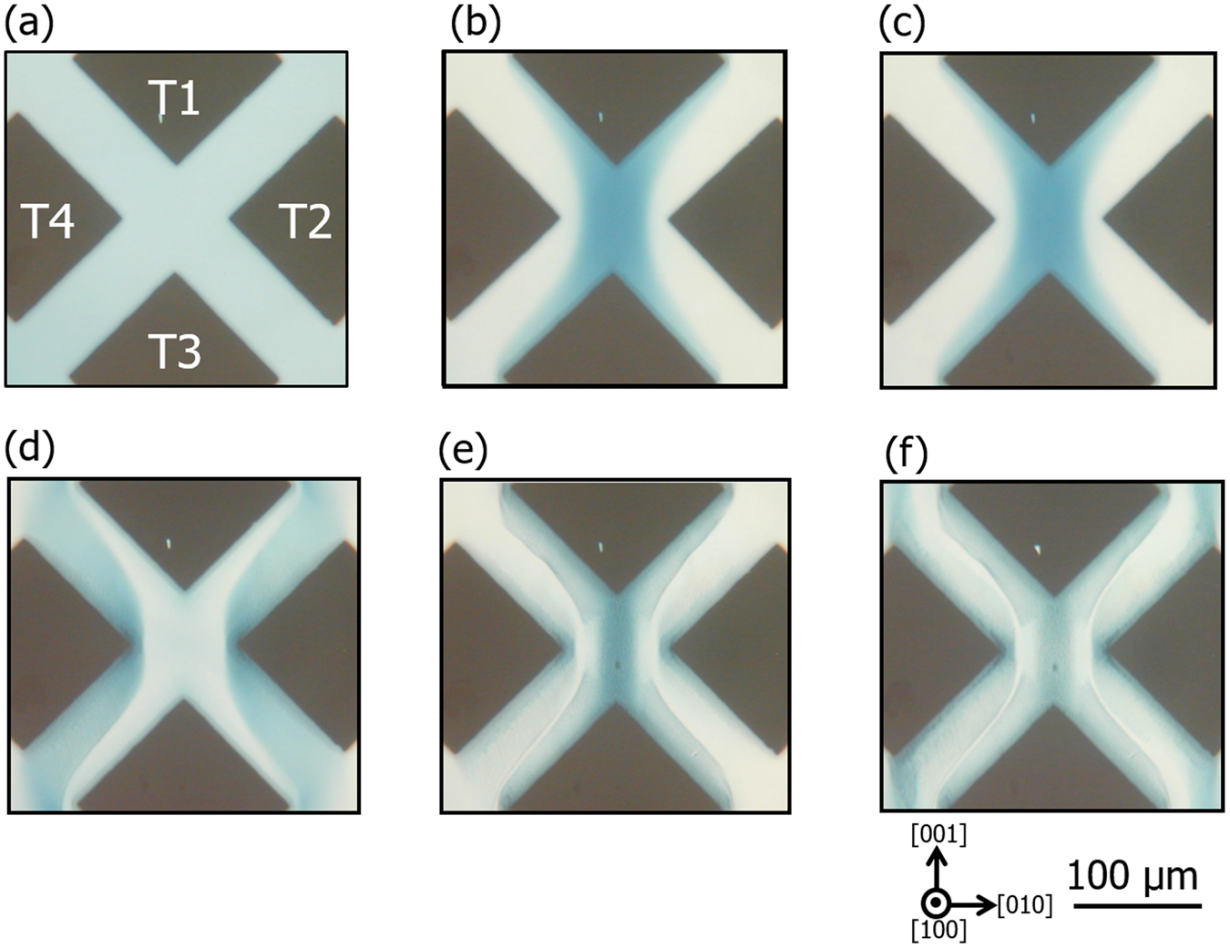
Representative optical microscope images showing internal structure of (100) device corresponding to measurement stage of *I*_1–3_ and *I*_2–4_ at cumulative times of *T*_*C*_ = (**a**) 0, (**b**) 500, (**c**) 950, (**d**) 1000, (**e**) 1100, and (**f**) 1150 s.

To obtain insight into the correlation between this alternate current variation and the oxygen vacancy distribution, the internal structure of the device was inspected using optical microscopy. In reduced TiO_2_, introducing oxygen vacancies produces trivalent titanium ions (Ti^3+^) that act as color centers. Therefore, change in the configuration of electro-coloring regions should reflect the change in the distribution (local concentration) of Ti^3+^ ions and oxygen vacancies, which is considered to be caused by drift motion of positively charged oxygen vacancies by an applied electric field. Figure 4(a–f) depict a series of representative transmission-mode optical micrographs of the device subjected to *V*_*C*_ for cumulative times of 0, 500, 950, 1000, 1100, and 1150 s, respectively, the observation stages of which are marked by red solid circles in Fig. 3. At the initial stage, the TiO_2−x_ area was faintly colored light blue, as shown in Fig. 4(a), after which an area colored dark blue appeared to bridge T1 and T3 while the other areas became colorless after application of *V*_*C*_ = +8 V for 500 s (Fig. 4(b)). This type of coloration in the TiO_2−x_ crystal is attributed to the electrocoloring phenomenon and the configuration of the electrocolored areas is modified by the application of an external voltage to the device. Therefore, when a positive voltage of *V*_*C*_ is applied to T2 and T4 under the condition where T1 and T3 are grounded, positively-charged oxygen vacancies are likely repelled from T2 and T4, and then accumulate around T1 and T3 due to drift motion induced by the electric field. The region colored dark blue contains a higher concentration of oxygen vacancies and is likely more conductive, whereas the colorless region has a lower concentration of oxygen vacancies and is less conductive. Thus, the configuration with T1 and T3 connected to each other through a zone with a high concentration of oxygen vacancies, i.e., high conductivity, accounts for the observed increase in *I*_1–3_. In contrast, T2 and T4 are observed to be surrounded by colorless areas; therefore, the resistance between them is determined by resistive areas with less conductivity, which results in a decrease in *I*_2–4_ compared with the initial value.

This type of bridge structure was then maintained until a time of 950 s (Fig. 4(c)), which is consistent with the lack of significant changes in *I*_1–3_ and *I*_2–4_ shown in Fig. 3. In contrast, as shown in Fig. 4(d), application of an additional −8 V for 50 s (cumulative time of 1000 s) made this structure completely inverted, where a colorless area bridged T1 and T3, while the other areas were colored dark blue. These features are also consistent with the observed *I*_1–3_ decrease; T1 and T3 are connected through a resistive region with a low oxygen vacancy concentration. On the other hand, the observed increase in *I*_2–4_ is likely due to a substantial increase in the areas where the T2 and T4 Pt electrodes are observed to be surrounded by a dark blue conductive region. These changes at the cumulative time of 1000 s look rather abrupt and discontinuous to be brought by the redistribution of oxygen vacancies under the constant voltage application. This intriguing behavior of abrupt switching is difficult to understand solely by considering field driven drift motion of the vacancies. One possible explanation for this is increased sample temperature by Joule heating^2,26^, which may strongly affect (promote) the migration and redistribution of vacancies after some incubation time. After subsequent applications of *V*_*C*_ at + 8 V and −8 V, as shown in Fig. 4(e,f), the contrast was inverted repeatedly, displaying features of dark blue and faint blue T1–T3 bridges, which correspond to the measured high and low *I*_1–3_ values, respectively. It should be noted that the colored structures become complicated and the degree of contrast inversion is reduced. The remaining faint blue contrast reflects the slightly higher *I*_1–3_ value measured at 1150 s compared with that at 1000 s, as shown in Fig. 3(a).

**Figure 5:**
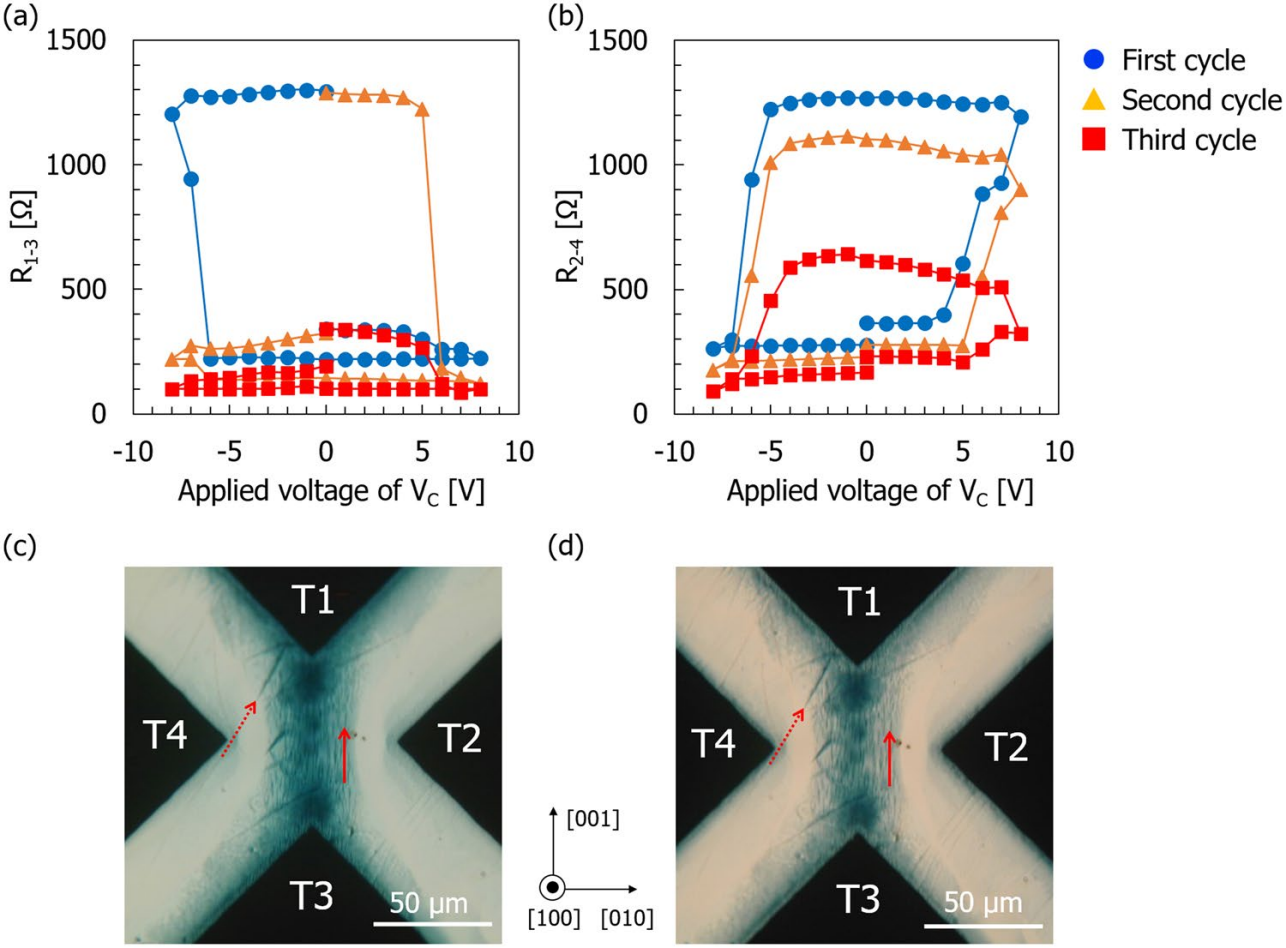
(**a,b**) Variations of resistance between T1 (T2) and T3 (T4) as function of applied voltage *V*_*C*_ in device fabricated on TiO_2−x_ (100) substrate. (**a**) *R*_1–3_ and (**b**) *R*_2–4_. (**c**,**d**) Optical micrographs showing internal structure of (100) substrate device surface. Images were obtained at *V*_*C*_ = (**c**) +8 V and (**d**) −8 V during the second cycle measurement. Solid and dotted arrows indicate positions of dark lines elongated along [001] and <011> directions, respectively.

Next, to examine the RS reversibility in the four-terminal devices, electrical measurements were implemented by applying the sequence shown in Fig. 2(b). Figure 5(a,b)) shows representative variations of resistance between T1 (T2) and T3 (T4), *R*_1–3_ (*R*_2–4_) as a function of the applied *V*_*C*_ in the device fabricated on the TiO_2−x_ (100) substrate. Here, the maximum and minimum *V*_*C*_ values were set to +8 V and −8 V, respectively, with a *T*_*C*_ of 100 s and a cycle where positive and negative *V*_*C*_ were applied was repeated three times. Note that once the *R*_1–3_ value is decreased, the *R*_2–4_ value is increased, and vice versa when the polarity of *V*_*C*_ is inverted, which indicates that the variations dependent on *V*_*C*_ are complementary to each other. Specifically, in the first cycle with an increasing *V*_*C*_ value, *R*_1–3_ (*R*_2–4_) is gradually decreased (increased) to the value at the positive maximum *V*_*C*_, so that the LRS (HRS) is achieved. The LRS (HRS) is stable while decreasing *V*_*C*_ but a sharp increase (decrease) in *R*_1–3_ (*R*_2–4_), a so-called RESET (SET) operation of the device, occurs at −7 V, so that the HRS (LRS) is achieved. The HRS (LRS) is then stable again while increasing *V*_*C*_. In the second and third cycles, the initial values of *R*_1–3_ and *R*_2–4_ start from the same values as those measured last in the previous cycle, and the trends of the *R*_1–3_ and *R*_2–4_ variations are almost similar to the first cycle. However, the maximum values of *R*_1–3_ and *R*_2–4_ in each cycle are observed to decrease with the measurement cycle, and this trend is much more obvious for *R*_1–3_ than for *R*_2–4_.

Microscopic observation of the device was performed to clarify the origin of this anomalous resistance change during the cyclic application of *V*_*C*_. At the stages of *V*_*C*_ = +8 V and −8 V in the first cycle, the characteristic oxygen vacancy distribution (data not shown) was confirmed to be quite similar to those in Fig. 4(b,d), respectively. In contrast, the internal structures of the device subjected to the second and third cycles did have distinct features. Figures 5(c,d) show transmission-mode optical micrographs of the device at the stages of *V*_*C*_ = +8 V and −8 V in the second cycle, respectively. Comparison with the first cycle case indicates the presence of additional dark line contrast elongating mainly along the [001] direction, as well as along <011> directions in the zone bridging between T1 and T3 (see arrows in Fig. 5). In particular, these dark lines still remain in the T1-T3 bridge, as shown in Fig. 5(d), although the dark blue behind them becomes faint due to the application of negative *V*_*C*_.

**Figure 6:**
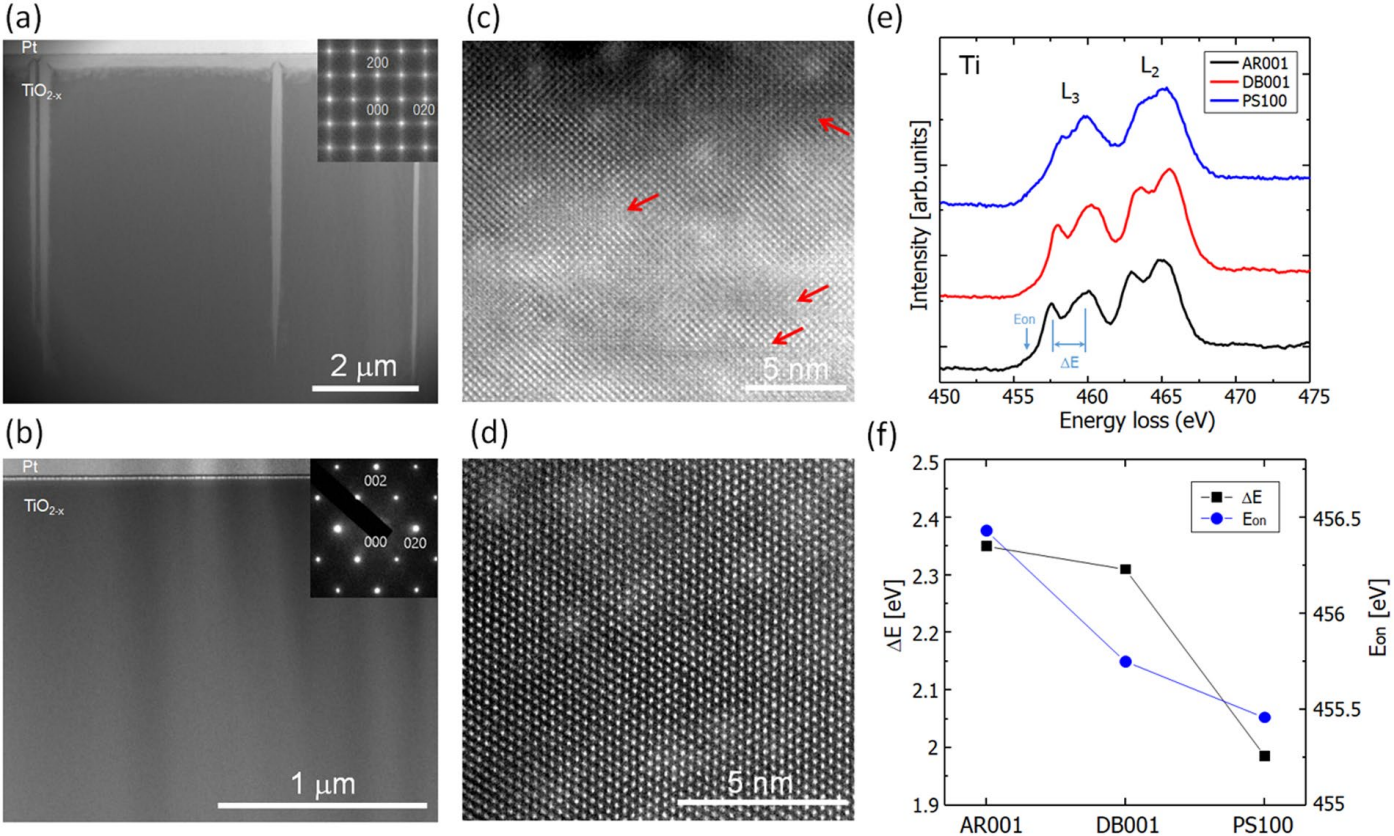
(**a**–**d**) Cross-sectional HAADF-STEM images and SAED patterns (insets) for electrically active zones in TiO_2−x_ four-terminal devices. (**a**,**c**) (100) device and (**b**,**d**) (001) device. Red arrows in (**c**) show the stacking disordered regions. (**e**) Ti L_2,3_ edge EELS spectra of electrically active zones in the different devices. (**f**) Energy onset *E*_on_, and e_g_-t_2g_ peak splitting Δ*E*, of Ti L_3_ edge. AR001, PS100, and DB001 denote as-reduced TiO_2−x_, PS in (100) device, and dark-blue regions in (001) device, respectively.

Scanning transmission electron microscopy (STEM) analysis demonstrated that these dark lines are closely related to shear plane defects in the TiO_2−x_ matrix, which result from a local accumulation of excess oxygen vacancies. Figure 6 shows cross-sectional high-angle annular dark field (HAADF)-STEM images acquired from the electrically active zones in the four-terminal devices. As shown in Fig. 6(a), high-contrast bright planar structures (PS) were observed in cross-section extending along the [$\bar{1}00$] depth direction, as well as the [010] direction in the vicinity of the surface of the (100) plane device, both of which correspond to the dark lines shown in Fig. 5. The selected area electron diffraction (SAED) pattern for the PS shows diffuse streaks along the <100> and <010> directions, as well as spots that correspond to single-crystal rutile TiO_2−x_, which indicates the existence of stacking disorders along these directions (Fig. 6(a) inset). This was verified from the high resolution HAADF-STEM image shown in Fig. 6(c), which shows the atomic arrangements locally disturbed in the form of shear plane defects^31^ in the single-crystal matrix. The Ti-L edge electron energy loss spectrum (EELS) acquired at the PS in the (100) device (PS100) is shown in Fig. 6(e). The spectrum for the PS shows a clear difference from that for the as-reduced TiO_2−x_ (AR001), especially in the shape and position of the L_3_ peaks. In addition, both the onset energy of the Ti-L_2,3_ edges (*E*_on_) and the energy splitting between the e_g_ and t_2g_ orbital peaks in the L_3_ edges (Δ*E*) for the PS show smaller values than those for AR (Fig. 6(f)), which suggests that the TiO_2−x_ phases in the PS turn into a further reduced state.

These changes in the atomic and electronic structures possibly occur as a result of oxygen vacancy accumulation caused by application of an external voltage to the device. In TiO_2_-based memristors, shear plane defects or the Magnéli phases are well known to act as highly conductive current paths, i.e., a conductive filament^24,25^. Furthermore, recent studies using density functional theory have predicted that the ordered arrangement of oxygen vacancies along the [001] direction is thermodynamically favorable and forms a conductive filament in rutile TiO_2_^32,33^. Thus the observed PS possibly reflects current leakage paths that are additionally formed in the T1–T3 bridge during application of the positive *V*_*C*_. Such leakage paths are not decomposed, even after application of the negative *V*_*C*_, so that the resistance of the T1–T3 bridge gradually decreases as the *V*_*C*_ cycle is repeated. This phenomenon eventually tends to deteriorate the controllability of the RS properties. Detailed analytical results of the crystallographic and electronic structures in the electrically active zone of these TiO_2−x_ memristive devices are also reported elsewhere^34^.

**Figure 7:**
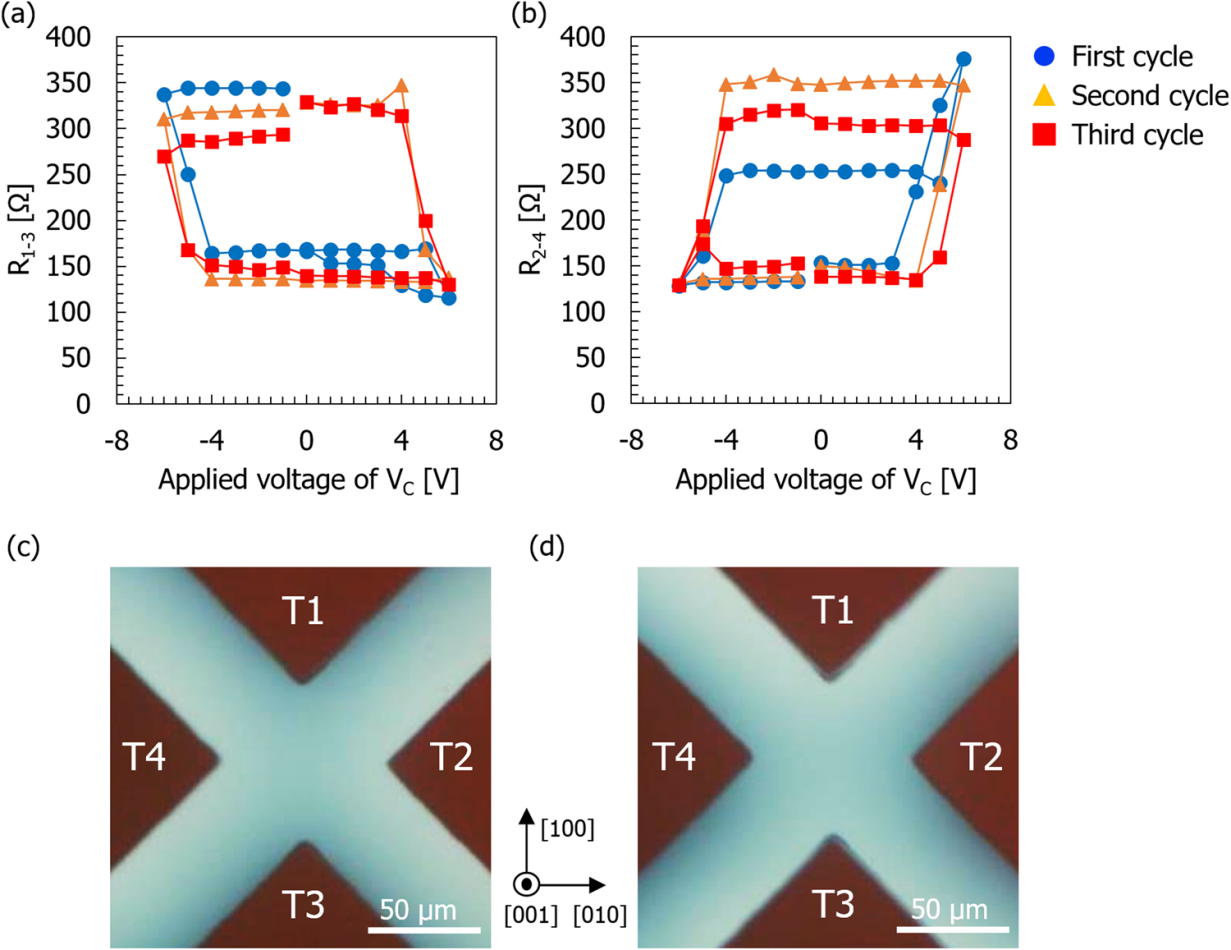
(**a**,**b**) Variations of resistance between T1 (T2) and T3 (T4) as function of applied voltage *V*_*C*_, in device fabricated on TiO_2−x_ (001) substrate. (**a**) *R*_1–3_ and (**b**) *R*_2–4_. (**c**,**d**) Optical micrographs showing internal structure of $(001)$ device surface. Images were obtained at *V*_*C*_ = (**c**) +6 V and (**d**) −6 V during the first cycle measurement.

The (100) substrate case shows the crude reversibility of RS against cyclic *V*_*C*_ application, which is mainly attributed to the irreversible structural change in the electrically active zone. In contrast, we have confirmed that much higher RS reversibility can be obtained for the device fabricated on the (001) substrate. Typical results for the variation of *R*_1–3_ and *R*_2–4_ as a function of the applied *V*_*C*_ in the (001) substrate device are shown in Fig. 7(a,b), respectively. Although some peculiar changes in both resistances were observed with an increase in *V*_*C*_ at the initial stage of the first cycle, the variation of *R*_1–3_ (*R*_2–4_) exhibits an orderly clockwise (counterclockwise) rotation as the *V*_*C*_ cycle is repeated. Furthermore, the threshold voltages for SET and RESET operations can be unambiguously determined — ±4 V in this case — and the maximum and minimum values of the resistances did not change significantly, even after the third cycle. These characteristics explicitly demonstrate the superiority of the (001) substrate device to the (100) device in terms of the stability of the RS properties.

Inspection of the oxygen vacancy distribution was also conducted and the results are shown in Fig. 7(c,d), where the inner structures are displayed for the device at the stages subjected to application of *V*_*C*_ at +6 V and −6 V in the first cycle, respectively. The interfaces between the dark blue and colorless areas are much broader than those evident for in the (100) device shown in Fig. 4. Figure 7(c) also shows that the dark blue region is constricted at the center between T2 and T4 by a diluted blue color. Comparison of this feature and those shown in Fig. 4 clearly indicates that the drift and diffusion behavior of oxygen vacancies is significantly dependent on the crystal orientation of TiO_2−x_ and the direction of the electric field applied to the device. The oxygen vacancy distribution shown in Fig. 7 accounts for the observed current variations, i.e., the formation of the dark-blue-colored region bridging T1 and T3 at *V*_*C*_ = +6 V corresponds to the lowest *R*_1–3_ (highest *R*_2–4_) measured, and this configuration is flipped to bridge between T2 and T4 at *V*_*C*_ = –6 V where the highest *R*_1–3_ (lowest *R*_2–4_) was measured. This type of pattern flipping was confirmed to be repeated in the second and third cycles. The irreversible structures, such as the dark line contrasts as observed in the (100) device, are not formed in the electrically active zone in this case. The absence of distinct structural changes in the crystal was also confirmed by STEM observations. The low magnification STEM image in Fig. 6(b) shows no significant features in the dark-blue-colored region of the electrically active zone of the (001) device (DB001). The SAED pattern and the high-resolution STEM image confirmed a single-crystal rutile structure, as shown in Fig. 6(b,d). These observations are in clear contrast to the case for the (100) devices where strong perturbation in the crystal structure was observed. However, the Ti-L edge EELS spectrum acquired for the DB001 device did indicate some changes compared with those for as-reduced TiO_2−x_: the onset energy *E*_on_ and the L_3_ peak splitting Δ*E* have lower values than those for the AR001 device (Fig. 6(f)), which indicates that the region is locally more reduced due to voltage application. It was thus deduced that this change in the electronic structure without a distinct change in the crystalline structure is the origin of the reversible RS characteristics in the (001) device.

**Figure 8:**
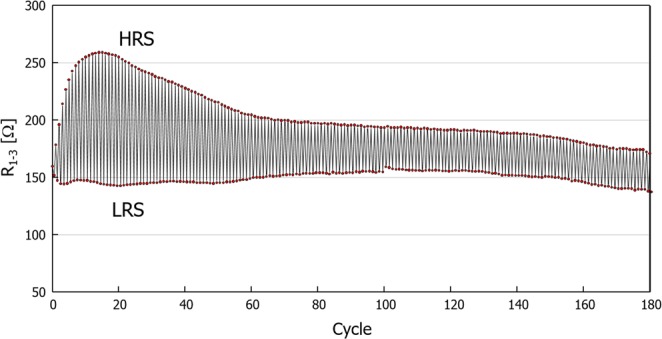
Endurance test result for (001) substrate device up to 180 cycles. *V*_*C*_ of ±6 V with *T*_*C*_ of 5 s was applied to switch the device between LRS and HRS.

Finally, a demonstration of the repeatability of the RS phenomenon, i.e., an endurance test for the memristive device fabricated on the (001) substrate is presented in Fig. 8. LRS and HRS can be switched more than 150 times in a stable manner, which ensures the stable operation of memristive devices based on carrier conductivity modulation controlled by modification of the oxygen vacancy distribution in the TiO_2−x_ crystal substrate. Concerning the reproducibility of the data, we confirmed that all the results presented here are reproducible in several times experiments. While at the same time, we observed some scattering and deviations in detailed behavior of individual devices, which may be attributed to local inhomogeneity of oxygen vacancy concentration caused by the initial crystal reduction process. One possible remedy for this is more precise control of the crystal (non-)stoichiometry based on thin film processes, and work along these lines are currently underway.

## Summary and Conclusions

We have demonstrated RS on the basis of carrier conductivity modulation caused by modification of the oxygen vacancy distribution in four-terminal planar devices fabricated on rutile TiO_2−x_ single-crystal substrates. The electrocoloring phenomenon effectively facilitates visual observation of the oxygen vacancy distribution and local transitions driven by an external voltage. Thus, a one-to-one correspondence between the electrically controlled LRS/HRS and the morphologically controlled oxygen vacancy distribution in the device is unambiguous. STEM analysis revealed that the electrically active zone in the (100) device is highly reduced and the atomic arrangements are strongly perturbed. However, the region with LRS in the (001) device was only moderately reduced and no significant changes in the crystal structure were observed. The reversibility of the RS phenomenon is strongly dependent on the crystallographic orientation of the TiO_2−x_ crystal; the superior characteristics of the (001) device with respect to the (100) device are attributed to suppression of the formation of conductive filaments in the RS zone. As also suggested in the previous report^12^, the feasibility of resistance control by modification of the oxygen vacancy distribution is expected to lead to the implementation of multilevel resistance in memristive devices. This functionality is particularly required for realizing continuous weight of synaptic connection in devices for neuromorphic computing. Such functional design will be investigated in the near future. In addition, the unique characteristics of complementary RS in the four-terminal memristive device provide an opportunity to not only develop gate-tuning memristive devices but also to pave the way for new types of memory architectures.

## Methods

Undoped rutile TiO_2_ (100) and TiO_2_ (001) single crystal substrates (Shinkosha Co., Ltd) were thermally annealed at 700 °C for 6 h under a vacuum of 1.5 × 10^−6^ Pa to prepare TiO_2−x_ substrates. The resistivity of the thermally annealed samples was measured by the van der Pauw method and estimated to be 6.37 Ω·cm. Four-terminal planar devices were fabricated by depositing square Pt electrodes on the annealed substrate surface. Each electrode was confirmed in advance to have an ohmic contact with the substrate, which typically occurs for highly-doped semiconductors. All electrical measurements were performed in a tungsten needle probe station installed in a vacuum chamber with a base pressure that was below 10^−3^ Pa using a semiconductor device analyzer (Keysight B1500A).

The internal structure of the devices was inspected using an optical microscope (Olympus BH2-UMA) after each measurement stage of *I*_1–3_ and *I*_2–4_. In addition, high angle annular dark-field (HAADF) imaging and electron energy-loss spectroscopy (EELS) were carried out using a C_S_ corrected scanning transmission electron microscope (STEM, JEOL ARM-200F system, 200 kV) to investigate the microscopic crystalline and electronic structures of the electrically active zone of the four terminal devices. EELS measurements were performed using a Gatan Quantum spectrometer and the spectra were recorded with a dispersion of 0.05 eV/channel. The energy resolution of EELS, i.e., the full width at half maximum (FWHM) of the zero-loss peak, was approximately 0.5 eV.
